# Rural-urban differences in accessing mental health care in Tunisia

**DOI:** 10.1192/j.eurpsy.2022.1616

**Published:** 2022-09-01

**Authors:** E. Bergaoui, M. Zrelli, N. Staali, M. Moalla, R. Lansari, A. Larnaout, W. Melki

**Affiliations:** Razi Hospital, Psychiatry D, Manouba, Tunisia

**Keywords:** Urban, mental healthcare access, Rural

## Abstract

**Introduction:**

Despite improvement of mental health outcomes over the last years in Tunisia, there are still striking rural-urban mental health inequalities.

**Objectives:**

The aim of this study is to evaluate the rural-urban differences in accessing mental health care among patients with psychiatric disorders

**Methods:**

A cross sectional and descriptive survey was conducted between March and April 2021 in the department of psychiatry D of Razi Hospital including 70 patients admitted or treated as outpatients. The sex ratio was 1.

**Results:**

The participants were aged between 17 and 68. About 11.42% came from rural areas. In these areas, 75% percent had low income versus 30.64% in urban areas. (p=0.047) The percentage of celibacy in urban areas was 68.85% versus 37.5% in rural areas (p=0.042) No significant difference was observed between the level of education and living in rural or urban areas. There was no association between rural or urban origin and number of admissions or treatment adherence or use of cannabis. The mean time between symptoms onset and consulting was 8.51 years in rural areas versus 2 years in urban areas. Moreover, time between symptoms onset and admission was significantly associated with rural or urban origin (p=0.045). The mean duration was 13,33 years (±10) in rural areas versus 3.12 years (±4.13).

**Conclusions:**

Families living in urban areas had better income and would come to psychiatric hospital earlier. Therefore, we should help patients in rural areas access to mental health facilities for a better medical care.

**Disclosure:**

No significant relationships.

